# MRI-Based Grading of Clear Cell Renal Cell Carcinoma Using a Machine Learning Classifier

**DOI:** 10.3389/fonc.2021.708655

**Published:** 2021-10-01

**Authors:** Xin-Yuan Chen, Yu Zhang, Yu-Xing Chen, Zi-Qiang Huang, Xiao-Yue Xia, Yi-Xin Yan, Mo-Ping Xu, Wen Chen, Xian-long Wang, Qun-Lin Chen

**Affiliations:** ^1^ Department of Rehabilitation Medicine, The First Affiliated Hospital of Fujian Medical University, Fuzhou, China; ^2^ Department of Radiology, The First Affiliated Hospital of Fujian Medical University, Fuzhou, China; ^3^ Department of Radiology, Xiangyang Central Hospital, Affiliated Hospital of Hubei University of Arts and Science, Xiangyang, China; ^4^ Department of Bioinformatics, School of Basic Medical Sciences, School of Medical Technology and Engineering, Key Laboratory of Medical Bioinformatics, Key Laboratory of Ministry of Education for Gastrointestinal Cancer, Fujian Medical University, Fuzhou, China

**Keywords:** machine learning, magnetic resonance imaging, texture analysis, clear cell renal cell carcinoma, multi-layer perceptron algorithm

## Abstract

**Objective:**

To develop a machine learning (ML)-based classifier for discriminating between low-grade (ISUP I-II) and high-grade (ISUP III-IV) clear cell renal cell carcinomas (ccRCCs) using MRI textures.

**Materials and Methods:**

We retrospectively evaluated a total of 99 patients (with 61 low-grade and 38 high-grade ccRCCs), who were randomly divided into a training set (*n* = 70) and a validation set (*n* = 29). Regions of interest (ROIs) of all tumors were manually drawn three times by a radiologist at the maximum lesion level of the cross-sectional CMP sequence images. The quantitative texture analysis software, MaZda, was used to extract texture features, including histograms, co-occurrence matrixes, run-length matrixes, gradient models, and autoregressive models. Reproducibility of the texture features was assessed with the intra-class correlation coefficient (ICC). Features were chosen based on their importance coefficients in a random forest model, while the multi-layer perceptron algorithm was used to build a classifier on the training set, which was later evaluated with the validation set.

**Results:**

The ICCs of 257 texture features were equal to or higher than 0.80 (0.828–0.998. Six features, namely Kurtosis, 135dr_RLNonUni, Horzl_GLevNonU, 135dr_GLevNonU, S(4,4)Entropy, and S(0,5)SumEntrp, were chosen to develop the multi-layer perceptron classifier. A three-layer perceptron model, which has 229 nodes in the hidden layer, was trained on the training set. The accuracy of the model was 95.7% with the training set and 86.2% with the validation set. The areas under the receiver operating curves were 0.997 and 0.758 for the training and validation sets, respectively.

**Conclusions:**

A machine learning-based grading model was developed that can aid in the clinical diagnosis of clear cell renal cell carcinoma using MRI images.

## Introduction

Renal cell carcinoma (RCC) is the most common malignant kidney tumor, and the most common pathological type, accounting for 70–90%, is clear cell renal cell carcinoma (ccRCC) (1). The latest World Health Organization (WHO)/International Society of Urological Pathology (ISUP) grading system divides ccRCC into four grades, in which grades I and II are low-grade tumors with good prognosis while grades III and IV are high-grade tumors with poor prognosis (2, 3). Current studies have shown a relationship between the different nuclear grades of RCCs and the choice of surgical methods and prognosis (4, 5). Therefore, preoperative determination of the nuclear grade of ccRCC is valuable.

The pathological features of renal masses are frequently evaluated by preoperative percutaneous renal biopsy, but this invasive technique still suffers from low accuracy. This has prompted a search for non-invasive methods that can grade the tumors and aid clinicians in selecting optimal therapeutic regimens. Several studies have proposed the use of images generated by computed tomography (CT) or magnetic resonance imaging (MRI) for identification of potential biomarkers for tumor grading (6, 7). MRI images have the advantage of being free from ionizing radiation exposure and are capable of evaluating both the tumor morphology and the tumor microenvironment (8), but MRI itself is incapable of providing sufficient information for differentiating the grades of ccRCC by most radiologists. However, artificial intelligence can play an important role in interpreting MRI information in comprehensive ways by texture analysis. In this way, MRI images can provide quantitative statistical parameters by identifying subtle texture information not readily observable with the human eye (8). These parameters, rather than the original images, can be then used as the input features for machine learning algorithms to improve the sensitivity of medical imaging diagnosis, and they can also circumvent the requirement for large sample sizes in image-based deep-learning algorithms. At present, the use of magnetic resonance texture analysis (MRTA) to predict ccRCC grades is seldom reported (9–11). The purpose of this study was to explore the value of using MRI textures and machine learning algorithms for predicting the grade of ccRCCs before operations.

## Materials and Methods

### Clinical Data

This retrospective study was approved by our Hospital Authority Review Committee. The requirement for informed consent was waived because of the study’s retrospective nature. The analysis included patients who met the following standards hospitalized from July 2016 to January 2020 at the First Affiliated Hospital of Fujian Medical University.

The inclusion criteria were: (i) patients surgically confirmed with ccRCCs; (ii) patients who had undergone preoperative contrast-enhanced MRI (corticomedullary phase, nephrographic phase, and delayed phase) in our hospital within one week before operations; and (iii) patients with single lesions with short diameters of more than 1 centimeter measured on axial T2 weighted imaging.

The exclusion criteria were: (i) patients with MRI images with artifacts, such as respiratory movement or magnetic sensitivity; (ii) patients with long lesion diameters ≤ 1cm; (iii) patients with tumors presenting as obvious cystic degeneration (cystic degeneration portion >75%); and (iv) patients with preoperative puncture biopsy, interventions, or other treatments.

We enrolled 99 patients with histologically verified ccRCC. These patients included 61 cases with low-grade disease (4 grade I cases and 57 grade II cases) and 38 cases with high-grade disease (32 grade III cases and 6 grade IV cases). The low-grade group included 42 males and 19 females, while the high-grade group included 25 males and 13 females. All MRI images were exported from the Picture Archiving and Communication System (PACS) of our hospital.

### MRI Examination

All patients underwent a preoperative 3.0 Tesla MR (MAGNETOM Verio, Siemens, Germany) examination with the standard protocol using a phased-array body coil. Image acquisition sequences and parameters were as follows: (a) coronal half-Fourier acquisition single-shot turbo spin-echo (HAST) sequences (repetition time msec/echo time msec, 1400/91; field of view, 340×340 mm; matrix, 224×320; section thickness, 5mm; intersection gap, 1mm); (b) transverse T2-weighted single-shot fast spin-echo sequences (repetition time msec/echo time msec, 2000/91; field of view, 340×340 mm; matrix 224×320; section thickness, 3 mm; intersection gap, 0.8mm); (c) axial diffusion weighted imaging sequences (repetition time msec/echo time msec, 6000/73; field of view, 340×340; section thickness, 4 mm; intersection gap, 0.8mm; and two sets of b values: 50 and 800 sec/mm^2^); (d) transverse three-dimensional fat suppressed T1-weighted interpolated spoiled gradient echo (volumetric interpolated breath-hold examination, VIBE) sequences (repetition time msec/echo time msec, 3.92/1.39; field of view, 250×380 mm; matrix, 224×320; section thickness, 3mm; intersection gap, 0.6 mm). The VIBE sequences were performed prior to and three times after intravenous injection of gadopentetate dimeglumine (MultiHance, Bracco Sine, Shanghai, China; 0.1 mmol per kilogram of body weight) at a rate of 2 mL/sec with a power injector (Medrad, Warrendale, USA), followed by a 20 mL saline flush. Corticomedullary phase images were obtained approximately 40–50 seconds after administration of the contrast material using timing, nephrographic phase images were obtained at 80–100 seconds, and excretory phase images were obtained 3 minutes later.

### Placement of ROIs

All data were stored anonymously in the Digital Imaging and Communications in Medicine (DICOM) format. The largest cross-section of the tumor on the axial CMP images was first determined, and then images of the selected layer were imported into MaZda (version 4.6, http://www.eletel.p.lodz.pl/mazda/). The two-dimensional region of interest (ROI) was then delineated manually by an experienced radiologist (Ziqiang Huang), who was engaged in urogenital system imaging diagnosis and blinded to the nuclear grade of the ccRCCs. Note that the edge of the lesion segmentation had shrunk inward by 1–2 mm.

### Feature Extraction

The differences in image brightness and contrast were reduced by standardizing the gray scale of the images before texture extraction, so that the image gray scale was within the range of [µ−3σ, µ+3σ], where µ and σ represent the mean gray value and the standard deviation, respectively. The MaZda quantitative texture analysis software package was used to extract texture features, including the gray-scale histogram, co-occurrence matrix, run-length matrix, and gradient and autoregressive models. All 257 radiomics features were extracted from each ROI for each patient. Three feature data sets (Data 1, Data 2, and Data 3) were obtained from the same tumor images by segmenting the data three times.

### Reproducibility of Texture Features

The reproducibility of the texture features was evaluated by calculating the intra-class correlation coefficients (ICCs) of 257 texture features among the three feature datasets. Only features with an ICC value equal to or higher than 0.80, indicating excellent reproducibility, were included in further feature selection processes.

### Feature Standardization

Before model development, various features were first standardized to make them comparable using the ‘robustscale’ method in the Python package of scikit-learn (ver. 0.23.2, https://scikit-learn.org/) (12). The specific formula is as follows:


$$z_{i}=\frac{V_{i}-M}{\text{IQR}}$$

where *V_i_* is the original feature value, *M* is the median of the feature, and IQR is the interquartile range (the difference between the third quartile and the first quartile). The following logistic transformation was then performed to minimize the adverse effects of outliers on the stability of the classifier:


$$y_{i}=1/\left(1+e^{-4z_{i}}\right).$$

### Feature Selection

A random forest model was used to select features for model development using the Random Forest Classifier function provided by scikit-learn. A grid search algorithm was then executed to determine a set of hyperparameters using the “GridSearchCV” function provided by scikit-learn. The random forest parameters were the following: ‘class_weight’ = ‘balanced’; ‘max_features’ = ‘log2’; and the rest were default values. A random forest model was then fitted to the training set, and the model then assigned each feature an importance coefficient that represents the information gain for the specific feature, where a larger value indicates a greater importance of the feature. The number of features was determined by repeated iterations based on the accuracy of the model on the validation set, while keeping the number of features as small as possible. Finally, 6 features, namely Kurtosis, 135dr_RLNonUni, Horzl_GLevNonU, 135dr_GLevNonU, S(4,4)Entropy, and S(0,5)SumEntrp, with the largest importance coefficients were selected.

### Model Development

We used a multilayer perceptron algorithm (the MLP Classifier function in scikit-learn) to develop the classification model. The model parameters were the following: ‘activation’ = ‘relu’, and ‘solver’ = ‘lbfgs’, ‘learning_rate’ = ‘constant’ and ‘hidden_layer_sizes’ = ‘(229),’. The most important parameter was ‘hidden_layer_sizes’, which determines the number of hidden layers and the number of nodes in each hidden layer. In this work, we included only one hidden layer, which consisted of 229 nodes. The number of nodes was optimized by repeated iterations to achieve optimal accuracy on the validation set.

### Statistical Analysis

Univariate analyses were performed with SPSS version 22 (SPSS Inc.). In the training set, the continuous variables (age, tumor size) between low-grade and high-grade groups were analyzed with the Student’s *t* test or the Mann–Whitney *U* test. The Chi-squared test was used to analyze the categorical variables (gender) between the two groups. A *p* value less than 0.05 was considered statistically significant.

## Results

### Demographic Analysis

The baseline characteristics of the training and validation sets are presented in **Table 1**. The training set consisted of 70 patients with pathologically proven ccRCC lesions (low-grade ccRCCs: 3 grade I lesions and 40 grade II lesions; high-grade ccRCCs: 23 grade III lesions and 4 grade IV lesions). The validation set consisted of 1 grade I lesion, 17 grade II lesions, 9 grade III lesions, and 2 grade IV lesions. In the training set, the mean ages ± standard deviations of the low-grade and high-grade subgroups were 53.5 ± 11.5 years and 57.1 ± 10.9 years, respectively. No statistically significant differences were found for gender and age distribution between the low-grade and high-grade ccRCC groups (*p* = 0.751 and 0.124, respectively). The average tumor sizes were 4.0 cm and 6.1 cm, respectively, in the low-grade and high-grade subgroups, and the difference was statistically significant (*p*<0.001).

**Table 1:** Analysis of baseline data from patients with ccRCCs.

Characteristic	Low-grade group	High-grade group	Statistics	*P* value
Patients (*n*)	61	38	–	–
Age (mean ± SD, years)	53.5 ± 11.5	57.1 ± 10.9	-1.522	0.124
Gender			0.1	0.751
Male (*n*)	42	25		
Female (*n*)	19	13		
Tumor size (mean ± SD, cm)	4.0 ± 2.1	6.1 ± 2.9	-3.869	<0.001

### MRI Texture Analysis and Feature Selection

The MRI images of 99 ccRCC tumors were used to extract 257 texture features with the MaZda software package. The features included 7 histogram features, 220 gray co-occurrence matrix features, 20 run-length matrix features, 5 gradient features, and 5 autoregressive model features.

The ICC ranges of the histogram features, gray level co-occurrence matrix features, run-length matrix features, gradient features, and autoregressive model features were (0.968, 0.998), (0.828, 0.996), (0.880, 0.997), (0.934, 0.986), and (0.863, 0.984), respectively.

Upon obtaining stable texture features, we applied the RobustScale method to standardize the feature values in the training set, and we then carried out a logistic transformation on them to minimize the negative impact of outliers on the model development (see *Materials and Methods* for details). The same formulas were archived and later applied to the validation set. The 257 processed features were input into a random forest model and fitted on the training dataset, while the hyperparameters of the random forest model were optimized with the grid search method.

**Figure 1:**
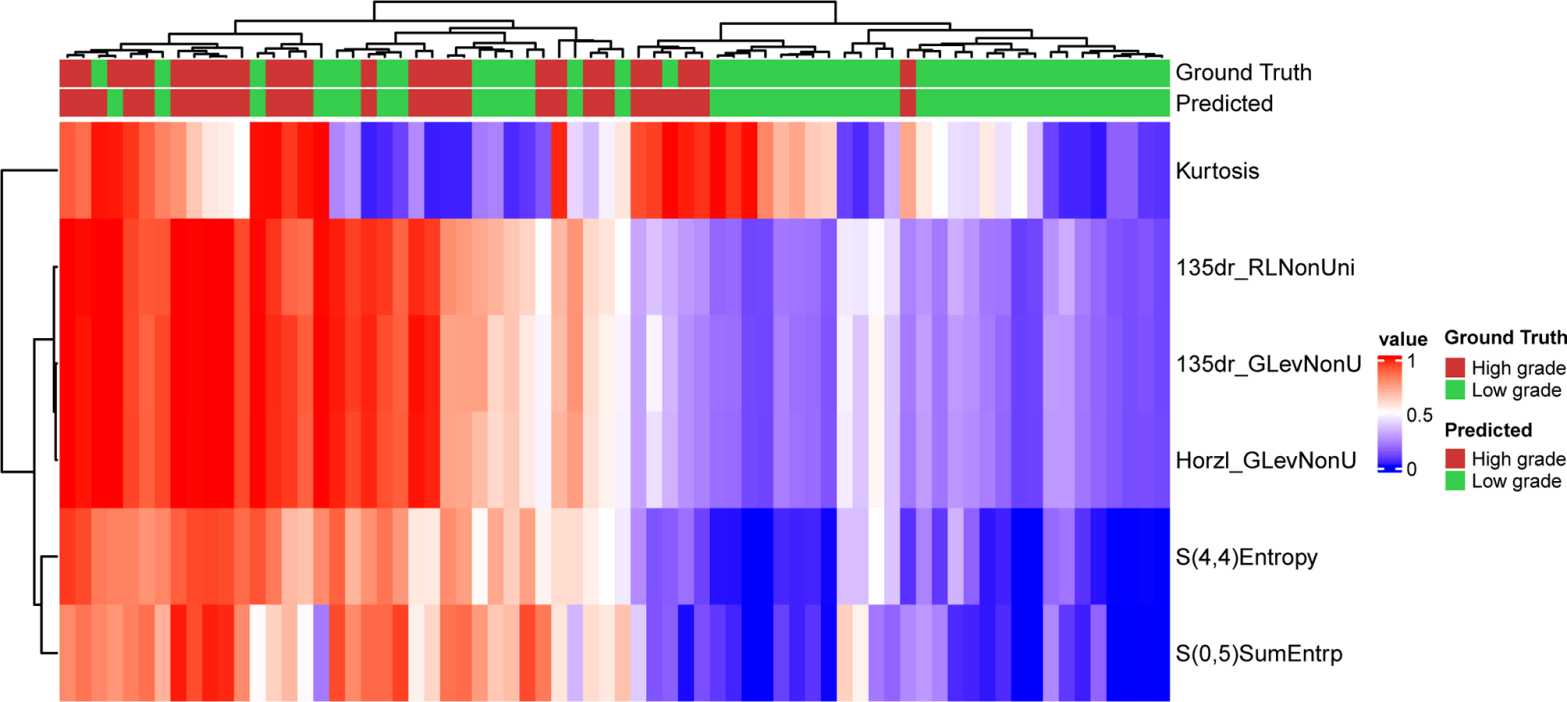
Prediction result and the heatmap of the selected 6 features in the training set along the clustering results of the samples and the features.

The model assigned an importance coefficient to each feature. The value of the coefficient represents the importance of the feature. A set of top-ranked probes was selected to develop the MLP model and to optimize the hyperparameters to achieve the highest accuracy in the validation set. With the optimized hyperparameters, the number of features was updated with the new fitting model. This iteration was repeated manually to obtain a minimal set of features without appreciably sacrificing the accuracy. In the final model, 6 texture features were selected for modeling. The heatmap of the 6 selected features is shown in **Figure 1** for the training set. **Figure 1** also shows that the low-grade and high-grade ccRCCs are approximately clustered into two separate groups, demonstrating the rationality of the selected features.

### Model Development

**Figure 2:**
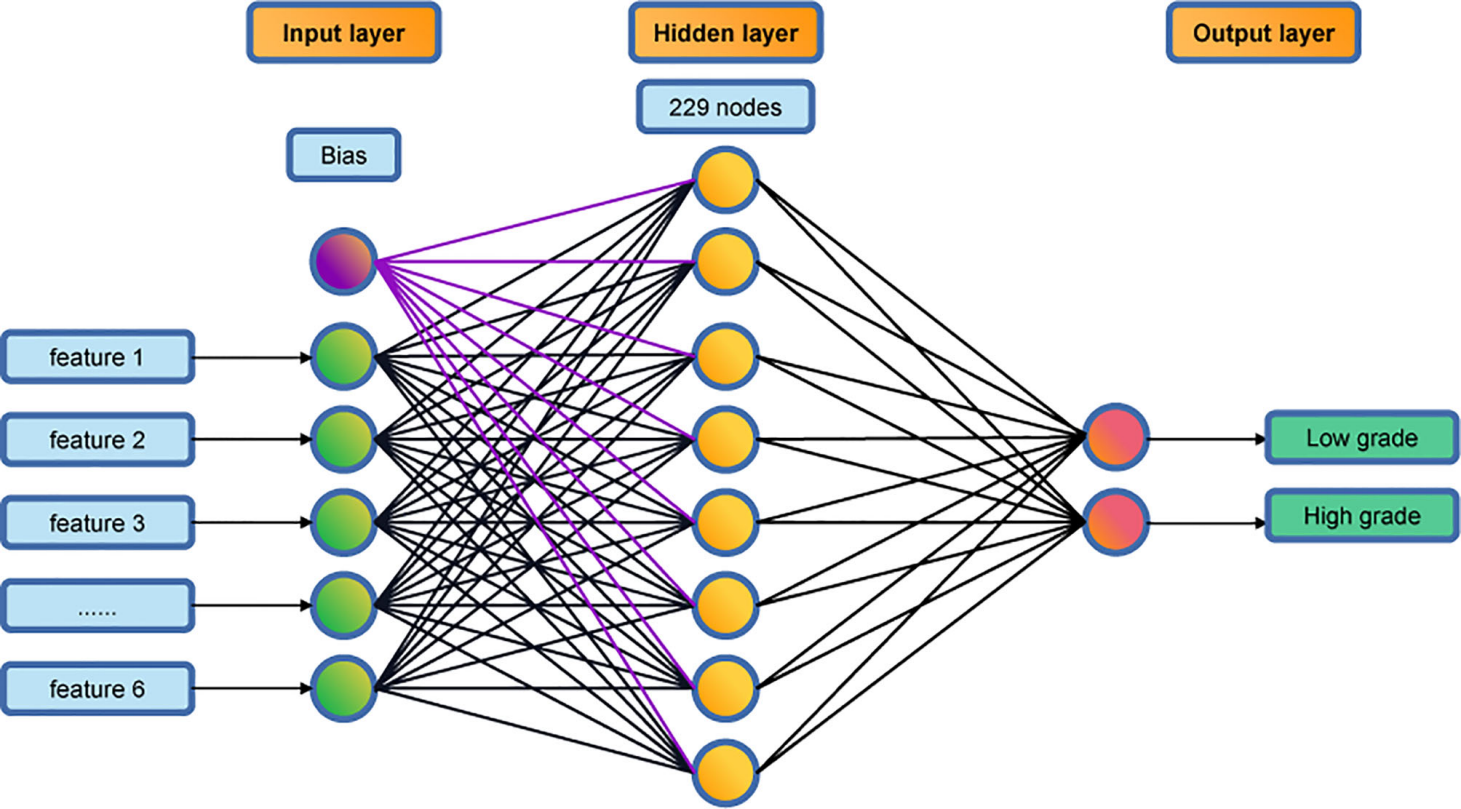
The topological structure of the 3-layer perceptron classifier.

A multi-layer perceptron algorithm was used for developing the prediction classifier. The final model has a three-layer structure: an input layer, a hidden layer, and an output layer (see **Figure 2**). The input layer consists of 6 nodes, corresponding to the 6 texture features, and the output layer consists of 2 nodes, corresponding to the low-grade and high-grade groups. The hidden layer has 229 nodes in the final model.

### Model Validation

**Figure 3:**
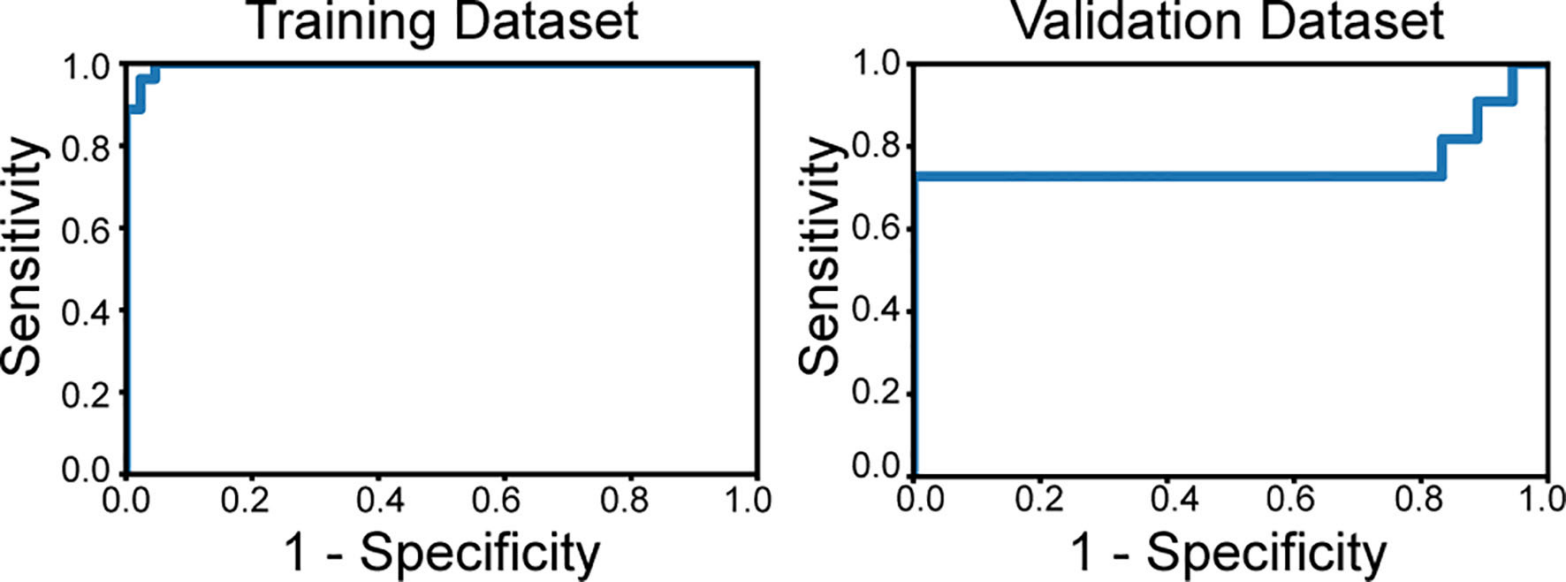
The receiver operating characteristics curves of the classifier applied to the training set and validation set. The area under curve is 0.997 in the training dataset and 0.758 in the validation set.

**Figure 4:**
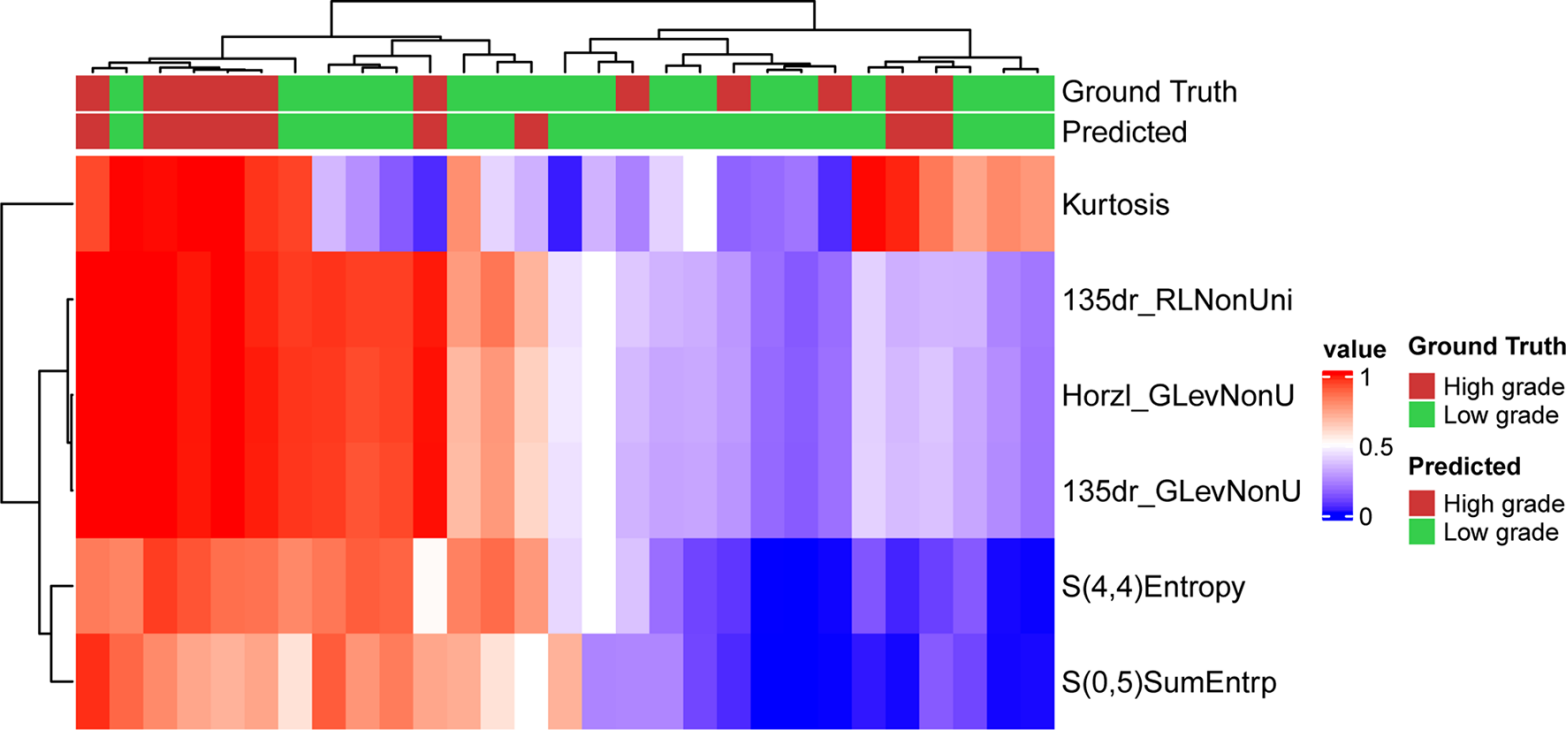
Prediction result and the heatmap of the selected 6 features in the validation set along the clustering results of the samples and the features.

**Figure 5:**
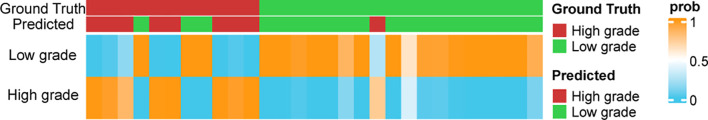
The prediction probabilities assigned by the classifier in the validation set. The top annotation labels show the ground truth and the predicted result.

The optimized model was evaluated in the validation set. The predictive indicators of the model in the training set and the validation set are shown in **Table 2**. The accuracy was 95.7% and 86.2%, respectively, in the training set and the validation set. The AUC values were 0.997 and 0.758, respectively, in the two sets (**Figure 3**). In the training set, two low-grade tumors were predicted as high-grade, and one high-grade tumor was incorrectly classified (**Figure 1**). In the validation set, only one low-grade and three high-grade tumors were misclassified (**Figure 4**). **Figure 5** shows that the misclassified low-grade tumors show higher similarities with the high-grade tumors and that, similarly, the misclassified high-grade tumors also show a higher similarity with the low-grade tumors. This result suggests that the selected texture features might be inadequate for discriminating these samples.

**Table 2:** Performance of the MLP classifier in the training and validation sets.

	AUC	ACC	SEN	SPE	PPV	NPV
Training set	0.997	0.957	96.30%	95.30%	92.90%	97.60%
Validation set	0.758	0.862	72.70%	94.40%	88.90%	85.00%

**Figure 5** shows the distribution of the probabilities predicted by the MLP model in the validation set. The prediction probability of the model’s prediction results for the 24/29 samples of the validation set is greater than 0.9, which indicates that the model is highly confident in the prediction result and is relatively robust.

## Discussion

In this study, we evaluated the applicability of a machine learning method based on MRI textures for the grade classification of ccRCCs. A three-layer MLP classifier using 6 features from MRI texture analysis exhibited satisfactory, reproducible, and reliable performance in discriminating the high-grade ccRCCs from the low-grade ones, and it outperformed classifiers presented in previous studies (8, 10, 13).

We adopted the latest WHO/ISUP grading system for renal cell carcinoma as the classifying criterion. However, most of the previous studies on the prediction of nuclear grading of ccRCC by texture analysis have been based on the Fuhrman classification system, which has some inevitable inadequacies, such as interpretation difficulties and poor reproducibility in clinical applications (10, 14). Besides the high application value for ccRCC, the WHO/ISUP nuclear grading is also a reliable prognosis indicator of patients with ccRCC (15).

In this study, we attempted to predict the nuclear classification of ccRCCs by quantitative analysis based on the texture features of MRI images. However, in current clinical practice, radiologists estimate the degree of aggressiveness of renal carcinoma based mainly on radiological findings (16, 17). For example, Pedrosa et al. found that some MRI features, which include both qualitative and semiquantitative parameters, can differentiate low-grade and high-grade ccRCCs (18). However, the classification is subjective and depends on the radiologist’s experience. Quantitative MRI texture analysis is now playing an increasingly important role in the clinical diagnosis and treatment of tumors, and it can be used to distinguish the pathological types and grades of tumors, to evaluate prognosis, and to predict the therapeutic response of tumors (19–21). Compared with CT examinations, MRI has multiple advantages, including multi-parameter imaging, high soft tissue resolution, high signal-to-noise ratio, and freedom from ionizing radiation. The texture features of multiple sequence images can be obtained with MRI, and this provides more feature space for developing imaging markers for tumors. Therefore, MRI texture analysis is a useful and promising method for non-invasive prediction of the ISUP nuclear grade of ccRCCs.

Multiple machine learning models have been successfully constructed to classify low-grade and high-grade ccRCCs (10, 13, 22). After comparing the performance of different models, we obtained an optimal prediction result with MLP. The AUC value of the classifier is 0.997 in the training set, indicating a good performance of the MLP model. Bektas et al. developed machine learning models to predict the Fuhrman nuclear grade of ccRCC based on quantitative CT texture analysis (22). They achieved the best prediction result using an MLP model with an AUC of 0.86. We further validated the application value of our model by creating a validation set to assess the accuracy and stability of the model. Satisfactory results were obtained, with an AUC of 0.758 in the validation set.

Most studies on machine learning-based CT or MRI texture analysis have not validated the developed models for predicting the nuclear grade of ccRCC. A comprehensive review of the radiomics literature on renal mass characterization in terms of validation strategies did not reveal any validation performed in 19 (46%) of the 41 papers reviewed (23). In other words, only slightly more than half of the studies described at least one validation method, and these were predominantly internal validation techniques. The wide clinical use of radiomics will require proper validation strategies for developing machine learning models. Compared with previous studies (24, 25), an independent and prospective test set is needed for further validation of our model in the future.

The current study has some inevitable limitations. One is that it is a single-center and retrospective study, so selectivity bias may exist. Another is the small sample size, which may lead to overfitting and low repeatability of the prediction results. Therefore, further expansion of the sample size and cross-verification of the model at multiple centers are needed. There is a slight imbalance in our dataset where the number of low-grade patients is larger than the number of high-grade patients. This issue could be addressed by the SMOTE algorithm (26). However, due the limited sample size we did not employ the method. Furthermore, the ratio between the sample size of the low-grade and that of the high-grade is approximately 3:2 where the class imbalance problem is not critical to the model performance. A third limitation is that the texture features extracted in this study are based on the two-dimensional ROI of MR images at the maximum level of the tumor, which may be biased by layer selection. Ideally, three-dimensional radiomic features of the whole lesion should be extracted to obtain comprehensive tumor features. A fourth limitation is that manual segmentation of MR images may be affected by the consistency between observers; however, this method is still widely used in texture analysis and remains the “gold standard” (27). Here, the stability of the texture features was evaluated by segmenting the lesions of all patients three times. Tumor size is generally associated with the tumor grade and therefore an important factor in tumor grading system. This information has been encoded in the feature “dr135RLNonUni” (Spearman’s ρ = 0.945 between the tumor diameter and dr135RLNonUni) and therefore it was implicitly used in the final model. The value of clinical factors other than radiomics signatures will also be investigated in predicting the grades in future study.

## Conclusions

An MLP model was successfully developed to classify the grades of clear-cell renal cell carcinomas, thereby demonstrating that ML-based MRI texture classifiers can be used preoperatively as a complementary tool to predict the ISUP grade of ccRCCs. This model can make a potential contribution to personalized treatment for patients with ccRCCs.

## Data Availability Statement

The raw data supporting the conclusions of this article will be made available by the authors, without undue reservation.

## Ethics Statement

Written informed consent was obtained from the individual(s) for the publication of any potentially identifiable images or data included in this article.

## Author Contributions

The work reported in the above for publications has been done by all authors. X-YC, YZ, and Y-XC contributed to data analysis, manuscript editing and model development and manuscript Preparation. Z-QH and X-YX collected the data of patients. Y-XY, M-PX, and WC helped in images analysis. X-lW and Q-LC helped in manuscript preparation and contributed to the supervision of the whole process. All authors contributed to the article and approved the submitted version.

## Funding

This work is supported by Fujian Medical University (Grant No. XRCZX2017001); the Natural Science Foundation of Fujian Province (Grant No. 2019J01294); Fujian Youth Education Research Project (Grant No. JT180193).

## Conflict of Interest

The authors declare that the research was conducted in the absence of any commercial or financial relationships that could be construed as a potential conflict of interest.

## Publisher’s Note

All claims expressed in this article are solely those of the authors and do not necessarily represent those of their affiliated organizations, or those of the publisher, the editors and the reviewers. Any product that may be evaluated in this article, or claim that may be made by its manufacturer, is not guaranteed or endorsed by the publisher.
